# Identification of *MAEL* as a promoter for the drug resistance model of iPSCs derived from T‐ALL

**DOI:** 10.1002/cam4.4712

**Published:** 2022-04-29

**Authors:** Xuemei Chen, Feiqiu Wen, Zhu Li, Weiran Li, Meiling Zhou, Xizhuo Sun, Pan Zhao, Chang Zou, Tao Liu

**Affiliations:** ^1^ Department of Tumor Immunotherapy, Shenzhen Luohu People's Hospital The Third Affiliated Hospital of Shenzhen University Shenzhen Guangdong P.R. China; ^2^ Medical Laboratory of Shenzhen Luohu People's Hospital Shenzhen China; ^3^ Department of Hematology and Oncology Shenzhen Children's Hospital Shenzhen China; ^4^ Department of Clinical Medical Research Center, The Second Clinical Medical College, Jinan University (Shenzhen People's Hospital) The First Affiliated Hospital of Southern University of Science and Technology Shenzhen Guangdong P.R. China

**Keywords:** drug resistance, IPSCs, *MAEL*, T‐ALL, transcriptome analysis

## Abstract

Significant progress has been made in the diagnosis and treatment of the drug‐resistant and highly recurrent refractory T cell acute lymphoblastic leukemia (T‐ALL). Primary tumor cell‐derived induced pluripotent stem cells (iPSCs) have become very useful tumor models for cancer research including drug sensitivity tests. In the present study, we investigated the mechanism underlying drug resistance in T‐ALL using the T‐ALL‐derived iPSCs (T‐iPSCs) model. T‐ALL cells were transformed using iPSC reprogramming factors (Sox‐2, Klf4, Oct4, and Myc) via nonintegrating Sendai virus. T‐iPSCs with the Notch1 mutation were then identified through genomic sequencing. Furthermore, T‐iPSCs resistant to 80 μM LY411575, a γ‐secretase and Notch signal inhibitor, were also established. We found a significant difference in the expression of drug resistance‐related genes between the drug‐resistant T‐iPSCs and drug‐sensitive groups. Among the 27 genes, six most differently expressed genes (DEGs) based on Log_2_FC >5 were identified. Knockdown analyses using RNA interference (RNAi) revealed that *MAEL* is the most important gene associated with drug resistance in T‐ALL cells. Also, *MAEL* knockdown downregulated expression of *MRP* and *LRP* in drug‐resistant T‐iPSCs. Interestingly, this phenomenon partially restored the sensitivity of the cells to LY411575. Furthermore, overexpression of the *MAEL* gene enhanced drug resistance against LY411575. Conclusively, *MAEL* promotes LY411575 resistance in T‐ALL cells increasing the expression of *MRP* and *LRP* genes.

## Graphical abstract

Our study investigated the mechanism underlying drug resistance in T‐ALL using the T‐ALL derived iPSCs (T‐iPSCs) model. We found that overexpression of MAEL promotes drug resistance in T‐ALL by increasing the expression of drug resistance‐related genes. Accordingly, MAEL is potentially a new treatment target for drug‐resistant T‐ALL.

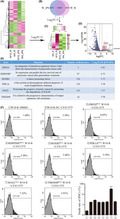

## 1 INTRODUCTION

Acute lymphoblastic leukemia (ALL) is a common hematological malignancy arising from abnormal proliferation and differentiation of hematopoietic stem cells. The etiology and pathogenesis of the disease are extremely complex.^1^ Mechanistically, T‐ALL arises from genetic alterations in the precursors of T‐cells, which arrest the development of the cells. This leads to the accumulation of blasts in the bone marrow, blood, thymus, and peripheral tissues.^2^ The deregulation of transcription factors, abnormalities in the regulation of the CDKN2A/2B cell cycle, and excessive activation of the NOTCH1 signaling have been implicated in the pathogenesis of T cell acute lymphoblastic leukemia (T‐ALL).^2^, ^3^ Although significant progress has been made in the diagnosis and treatment of T‐ALL, drug resistance and recurrence of T‐ALL remain major clinical concerns.^1^

Induced pluripotent stem cells (iPSCs) are developed by introducing pluripotent genes in somatic cells. The properties of iPSCs such as unlimited proliferation and multidirectional differentiation are similar to those of embryonic stem cells. iPSCs can differentiate into any of the three types of embryonic cells, which mirrors the developmental process of human embryonic tissues. In general, iPSCs play an invaluable role in the field of regenerative medicine,^4^, ^5^, ^6^, ^7^, ^8^, ^9^ blood transfusion,^10^, ^11^, ^12^ tumor immune cell therapy,^13^, ^14^ tumor vaccine development,^15^, ^16^, ^17^ drug researches,^18^ and disease modeling^18^, ^19^, ^20^ among others.

iPSCs derived from primary tumor cells have become very useful tools in modeling tumor diseases for various researches such as drug sensitivity testing. Acute myeloid leukemia (AML)‐derived iPSCs (AML‐iPSCs) can differentiate into hematopoietic stem cells in vitro ^21^, ^22^, ^23^. When transplanted into immunodeficient mice, the differentiated hematopoietic stem cells transform into aggressive myeloid leukemia. Epigenetic analyses have further revealed that reprogramming of AML cells changes DNA methylation in these cells. When AML‐iPSCs differentiate into hematopoietic stem cells, they undergo DNA methylation similar to that in primary AML cells.^23^ iPSCs derived from tumor cells can differentiate to corresponding lineages of tumor cells. Thus, iPSCs can be used as tumor models in vitro.^23^

Compared with the traditional cancer cell lines and in vivo cancer models, cancer‐derived iPSCs present unique advantages. First, the cancer‐derived iPSC model was a kind of individualized tumor model for patients.^24^ As such, they can be used for studying the early stages of tumor progression.^25^ Second, cancer‐derived iPSCs can differentiate into mature tumor cells. Genetic and epigenetic analyses have revealed mutations and epigenetic alterations that promote tumorigenesis.^25^ Third, cancer‐derived iPSCs can proliferate indefinitely. Accordingly, the cancer‐derived iPSC were the ideal models for the toxicity study.^24^ Finally, all carcinomas have the feature of heterogeneity. iPS clones developed from different tumor cells can be used to study the complex pathogenesis of tumor.^25^

In the present study, we investigated the mechanism underlying drug resistance in T‐ALL using T‐iPSCs. The findings of this study will provide new and more effective methods for the treatment of drug‐resistant and recurrent T‐ALL.

## 2 MATERIALS AND METHODS

### 2.1 Isolation and culture of PBMCs

Blood samples of T‐ALL patients were obtained from Shenzhen Children's hospital. According to the clinical data of patients, one case of T‐ALL harboring Notch1 mutation (NM_017617.3:c.5033 T>C [p.L1678P]) were selected to isolate PBMCs. Three days before transformation, PBMCs were isolated from fresh peripheral blood of volunteers using the Lymphoprep™ Density gradient medium (07861; Stemcell). The PBMCs were resuspended in StemSpan™ SFEM (9650; Stemcell) supplemented with 1% l‐Glutamine (25,030,081; Thermo), 100 ng/ml SCF (C034; Nanoprotein), 100 ng/ml FLT3LG (CA82; Nanoprotein), 20 ng/ml IL‐3 (CD90; Nanoprotein), and 10 ng/ml IL‐6 (C009; Nanoprotein) (PBMC medium for short). Subsequently, 1 × 10^6^ PBMCs were seeded into wells of a 24‐well plate and cultured under 5.0% CO_2_ at 37°C. Half of the culture medium was changed every day for 3 days.

### 2.2 Development of iPSCs from PBMCs

PBMCs were transformed using the CytoTune®‐iPS 2.0 Sendai Reprogramming Kit (A16517; Thermo Fisher Scientific), according to the manufacturer's protocol. Briefly, on day 0, 2 × 10^5^ of the cultured PBMCs were infected with Sendai virus at multiple of infection (MOI) of 5:5:3 (KOS: c‐Myc: Klf4). The mixture was then centrifuged at 1000 ***g*** for 30 min at room temperature. The PBMCs were resuspended in the supernatant again, inoculated into a 24‐well plate, and cultured under 5.0% CO_2_ at 37°C. After 24 h of infection, the PBMCs were collected and centrifuged at 200 ***g*** for 10 min at room temperature. The supernatant was discarded before resuspending the PBMCs in 0.5 ml PBMC medium. The PBMCs were transferred to a 24‐well plate and cultured under 5.0% CO_2_ at 37°C for 48 h. On day 3, the PBMCs were transferred to a six‐well plate containing 1 ml StemSpan™ SFEM medium without cytokines. The plate wells were pre‐coated with Laminin‐521 (LN521‐03; BioLamina). On day 4, 50% of the medium was replaced with NutriStem hESC XF medium. From day 6, the media was changed every day until embryonic stem cell‐like colonies were observed (day 12–21). T‐iPSCs (W4‐iPS and W10‐iPS) were generated from T‐ALL harboring Notch1 mutation.

### 2.3 Alkaline phosphatase staining

Alkaline phosphatase staining of iPSCs was performed using the VECTOR Blue Alkaline Phosphatase (AP) Substrate Kit (SK‐5300; Vector Laboratories), according to the manufacturer's instructions. Stained cells were observed and photographed under an inverted microscope (AF6000; Leica) at 4× objectives.

### 2.4 Immunofluorescence staining

Immunofluorescence staining of pluripotent markers in iPSCs was performed as previously described.^26^ The antibodies used included rabbit anti‐Sox2 antibodies (1:400, A11936; Abclonal), mouse anti‐SSEA4 antibodies (1:500, ab16287; Abcam), rabbit anti‐POU5F1 antibodies (1:400, A7920; Abclonal), mouse anti‐NANOG antibodies (1:500, YM0464; Immunoway), Alexa Fluor 488‐labeled goat anti‐rabbit IgG antibodies (1:500, A0423; Beyotime), and Alexa Fluor 647‐labeled goat anti‐mouse IgG antibodies (1:500, A0473; Beyotime). The antibodies were labeled with 4′,6‐diamidino‐2‐phenylindole dihydrochloride (DAPI) (Sigma‐Aldrich).

### 2.5 Formation and detection of teratoma

iPSCs generated by PBMCs were transplanted subcutaneously into NOD/SCID mice. The presence of teratoma was assessed using hematoxylin and eosin (H&E) staining. Briefly, 3 × 10^6^ to 5 × 10^6^ iPSCs were subcutaneously injected into healthy adult NOD/SCID mice aged 6–10 weeks (Guangdong Medical Laboratory Animal Centre, China). About 30–40 days after injection, the teratoma grew into a spherical mass of about 1–2 cm in diameter. The mass was resected, fixed in 10% neutral formalin buffer for 3 days, and stored in 100% ethanol. The mass was then processed for H and E staining. The typical structures of cells from three embryonic germ layers were then identified using a microscope.

### 2.6 Development of drug‐resistant T‐iPSCs

Drug‐resistant iPSCs were developed as previously described.^27^ Briefly, T‐iPSCs (W4‐iPS and W10‐iPS) were treated with varied dosages of LY411575 ranging from 1.25 to 160 μM for 72 h. The cell death rate was assessed using 7AAD staining. Half maximal inhibitory concentration (IC50) of LY411575 was then calculated. To develop drug‐resistant T‐iPSCs, T‐iPSCs were first incubated for 24 h with 1/6 IC50 of LY411575. T‐iPSCs that survived were cultured in a drug‐free medium for 3–5 days before the second cycle of drug treatment. After eight cycles of drug treatment, T‐iPSCs grew stably at this concentration. Then, the drug concentration was increased and the drug treatment process was repeated until T‐iPSCs could grow stably in 80 μM LY411575. The resistant W4‐iPS (W4‐R) and W10‐iPS (W10‐R) cells were cultured in iPSC medium supplemented with 80 μM LY411575.

### 2.7 RNA sequencing and bioinformatics analysis

Total RNA was extracted from W4‐R or W10‐R iPSCs using TRIzol (Thermo, 15596–026). Briefly, 1 × 10^6^ W4‐R or W10‐R iPSCs were lysed with 0.4 ml TRIzol Reagent. After incubation for 5 min, 80 μl chloroform was added. After centrifuged the sample for 15 min at 12,000 ***g*** at 4°C, the aqueous phase containing the RNA was transferred to a new tube. Then, the extracted RNA was precipitated with 200 μl isopropanol and washed with 400 μl 75% ethanol. At last, the total RNA was solubilized in 50 μl RNase‐free water.

RNA sequencing (RNA‐seq) was performed on Illumina HiSeq. The sequencing reads of RNA‐seq were aligned to the human hg19 genome using Hisat2 software with default parameters.^28^ Then, the reads count for each gene was extracted by featureCounts^29^ and used as input for DESeq2 R package^30^ for differential expressed gene analysis (DEG). Genes with log_2_FC ≥ 1 and FDR ≤ 0.05 were considered as DEGs. The *z*‐score normalized reads count of all differential protein coding genes was used to plot the heatmap and volcano figure with R.

### 2.8 RNA interference of W10‐R iPSCs

W10‐R iPSCs were transfected with siRNAs (Genepharm) against six genes (*ZBED2*, *SERPINB7*, *HOXB2*, *PDE1A*, *MAEL,* and *TMEM40*) using 100 nM Lipofectamine® 3000 (Thermo Fisher Scientific), according to the manufacturer's instructions. The transfected cells were cultured for 72 h before subsequent experiments. To avoid off‐target effects, three pairs of siRNAs were used. FAM‐labeled scrambled siRNA was used as the negative control. The sequences of siRNAs used in this study are shown in Table 1.

**TABLE 1:** Sequences of siRNAs

Number	Name	Sequences (5′ to 3′)
1	ZBED2‐Homo‐1075	UCUGAGGCAUGGGAAUAUUTT
		AAUAUUCCCAUGCCUCAGATT
	ZBED2‐Homo‐1119	GCACCAUCCCAACCAGUAUTT
		AUACUGGUUGGGAUGGUGCTT
	ZBED2‐Homo‐1476	CCUGGAGAUGAAGUGGAAGTT
		CUUCCACUUCAUCUCCAGGTT
2	SERPINB7‐Homo‐698	GCGAGUUGACUUUACGAAUTT
		AUUCGUAAAGUCAACUCGCTT
	SERPINB7‐Homo‐853	GCAAGUGGCAAUCAGCCUUTT
		AAGGCUGAUUGCCACUUGCTT
	SERPINB7‐Homo‐922	GGAAGGCAGUCGCCAUGAUTT
		AUCAUGGCGACUGCCUUCCTT
3	HOXB2‐Homo‐140	GGGAGAUUGGGUUUAUAAATT
		UUUAUAAACCCAAUCUCCCTT
	HOXB2‐Homo‐674	GGCAGGUCAAAGUCUGGUUTT
		AACCAGACUUUGACCUGCCTT
	HOXB2‐Homo‐903	GCCUUUAGCCGUUCGCUUATT
		UAAGCGAACGGCUAAAGGCTT
4	PDE1A‐Homo‐420	GGAAGCAGUUUAUAUCGAUTT
		AUCGAUAUAAACUGCUUCCTT
	PDE1A‐Homo‐856	GUUGGUUACAGCAAGUACATT
		UGUACUUGCUGUAACCAAACTT
	PDE1A‐Homo‐1190	GGAACCUAGUGAUUGAAAUTT
		AUUUCAAUCACUAGGUUCCTT
5	MAEL‐Homo‐944	GCGUACUGCAUCAGUAAUUTT
		AAUUACUGAUGCAGUACGCTT
	MAEL‐Homo‐1067	GGGCGUUACCAGAAGCUAATT
		UUAGCUUCUGGUAACGCCCTT
	MAEL‐Homo‐1148	CCCAUUGGUGACUACCCAUTT
		AUGGGUAGUCACCAUGGGTT
6	TMEM40‐Homo‐213	GCCAUGGAGACUUCAGCAUTT
		AUGCUGAAGUCUCCAUGGCTT
	TMEM40‐Homo‐304	UCCACAAGCAAGAUGGGAATT
		UUCCCAUCUUGCUUGUGGATT
	TMEM40‐Homo‐889	GGCUGACAGGGUUCAGGAATT
		UUCCUGAACCCUGUCAGCCTT
7	Negative control (FAM)	UUCUCCGAACGUGUCACGUTT
		ACGUGACACGUUCGGAGAATT

### 2.9 Quantitative real‐time polymerase chain reaction (qRT‐PCR)

The efficiency of knockdown of genes in W10‐R iPSCs was analyzed using qRT‐PCR. Total RNA from 1 × 10^6^ W10‐R iPSCs was extracted using Aurum™ Total RNA Mini Kit (Bio‐Rad). The RNA (1 μg) was reverse transcribed to cDNA using the BioSci™ WitEnzy First‐Stand cDNA Synthesis Kit (Dakewe). The BioSci™ WitEnzy 2 × SYBR Green qPCR Master Mix (Dakewe) was used for RT‐PCR reaction according to the manufacturer's instructions. Notably, *18sRNA* was used as the internal control. The sequences of primers used in the qRT‐PCR are shown in Table 2.

**TABLE 2:** Nucleotide sequences of the qRT‐PCR primers

Name	Sequences (5′ to 3′)	Fragment size (bp)
ZEBD2‐F	GGCAAAAGGGGACTTAGAGATG	84
ZEBD2‐R	GGCATAGCACTCACAAAAGGG	
H‐SERPINB7‐F	TAAGCTCATCTGCTGTAATGGTG	93
H‐SERPINB7‐R	GGCAATTTATGGTTTCGCTCTTG	
H‐HOXB2‐F	CGCCAGGATTCACCTTTCCTT	92
H‐HOXB2‐R	CCCTGTAGGCTAGGGGAGAG	
H‐PDE1A‐F	GCATACAGGGACAACAAACAAC	83
H‐PDE1A‐R	TCTCAAGGACAGAGCGATCAT	
H‐MAEL‐F	GAAGATCCCCGAACTACGGC	94
H‐MAEL‐R	GAAAACAGGTTTCGCCCAGTC	
H‐TMEM40‐F	CAGAGCAACCGGAAAACATCG	102
H‐TMEM40‐R	TCATCCTTCAAAACGTCAGGC	
MRP‐F	TGGGACTGGAATGTCACG	260
MRP‐R	AGGAATATGCCCCGACTTC	
LRP‐F	GTCTTCGGGCCTGAGCTGGTGTCG	240
LRP‐R	CTTGGCCGTCTCTTGGGGGTCCTT	
18sRNA‐F	AACTTTCGATGGTAGTCGCCG	
18sRNA‐R	CCTTGGATGTGGTAGCCGTTT	

### 2.10 MAEL‐GFP plasmid transfection and drug resistance analysis in W10‐iPS cells

The pcDNA3.1‐zeo‐EmGFP‐MAEL (MAEL‐GFP) plasmid was constructed by inserting the *MAEL* gene into pcDNA3.1‐zeo‐EmGFP. The genetic map of the MAEL‐GFP plasmid is shown in Figure S1. The MAEL‐GFP plasmid was then transfected into W10‐iPS cells using Lipofectamine 3000 (Thermo Fisher Scientific). After 72 h of transfection, 80 μM LY411575 was added to the culture system for another 72 h. W10‐iPS cells were then digested, stained with 7AAD and analyzed using flow cytometry to assess the rate of cell death.

### 2.11 Statistical analysis

The experiments were performed at least three times. Data were presented as mean ± standard deviation (SD). Differences between groups were analyzed using the one‐way analysis of variance (ANOVA). Statistical significance was set at *p* < 0.05.

## 3 RESULTS

### 3.1 Human T‐ALL cells could be transformed into iPSCs without altering the initial Notch1 mutation

**FIGURE 1:**
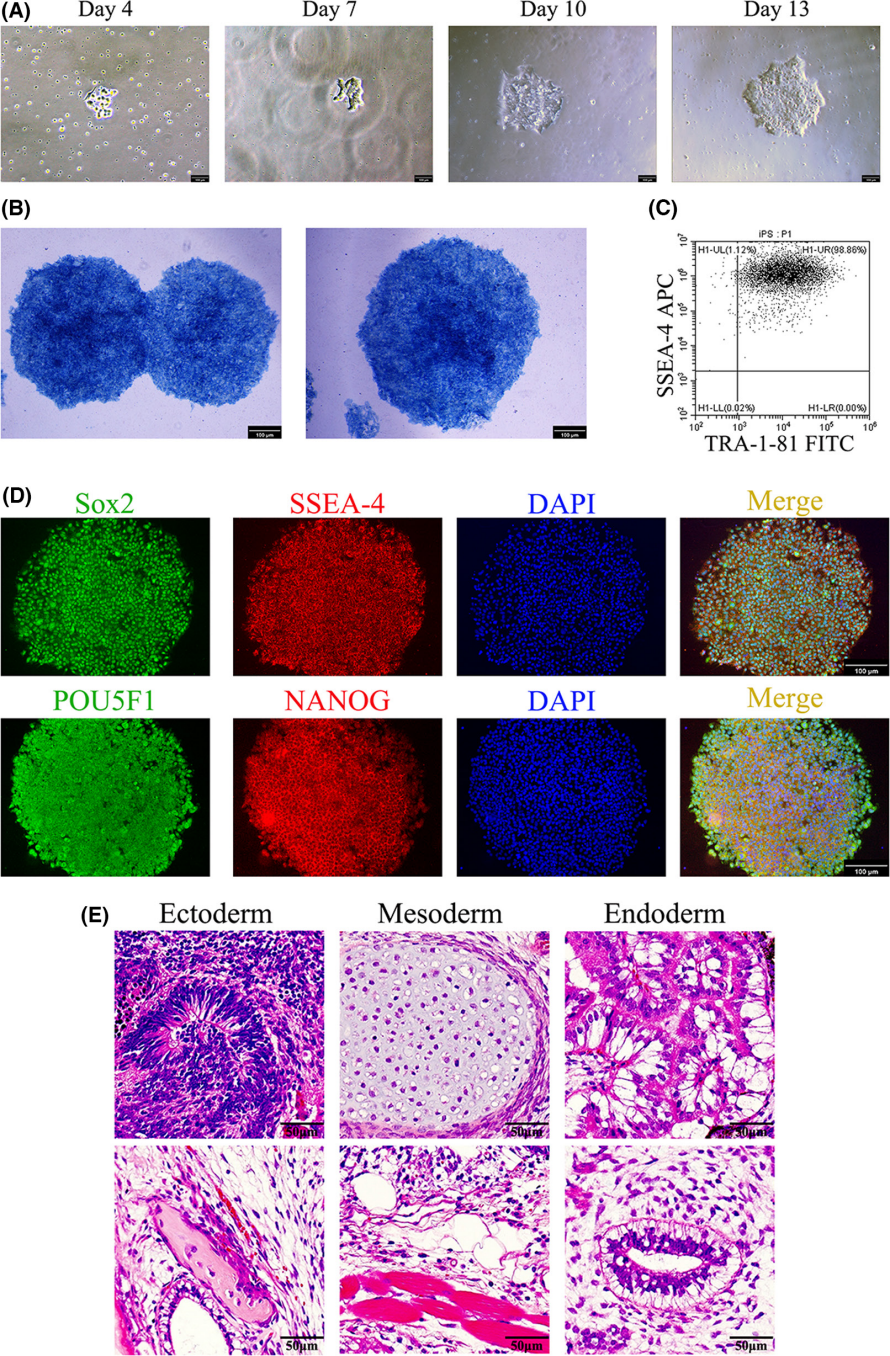
Reprogramming of human T‐ALL cells into iPSCs. (A) Representative clonal growth during reprogramming process of T‐ALL cells. The photos were captured using a standard Nikon microscope under 10× magnification. (B–D) Pluripotency analyses of iPSC clones. The analyses were performed using alkaline phosphatase staining (B), flow cytometry (C), and immunofluorescence staining (D). (E) H and E staining of iPSCs transplanted subcutaneously into NSG mice. The teratoma contained all three germ layers (ectoderm, mesoderm, and endoderm)

**FIGURE 2:**
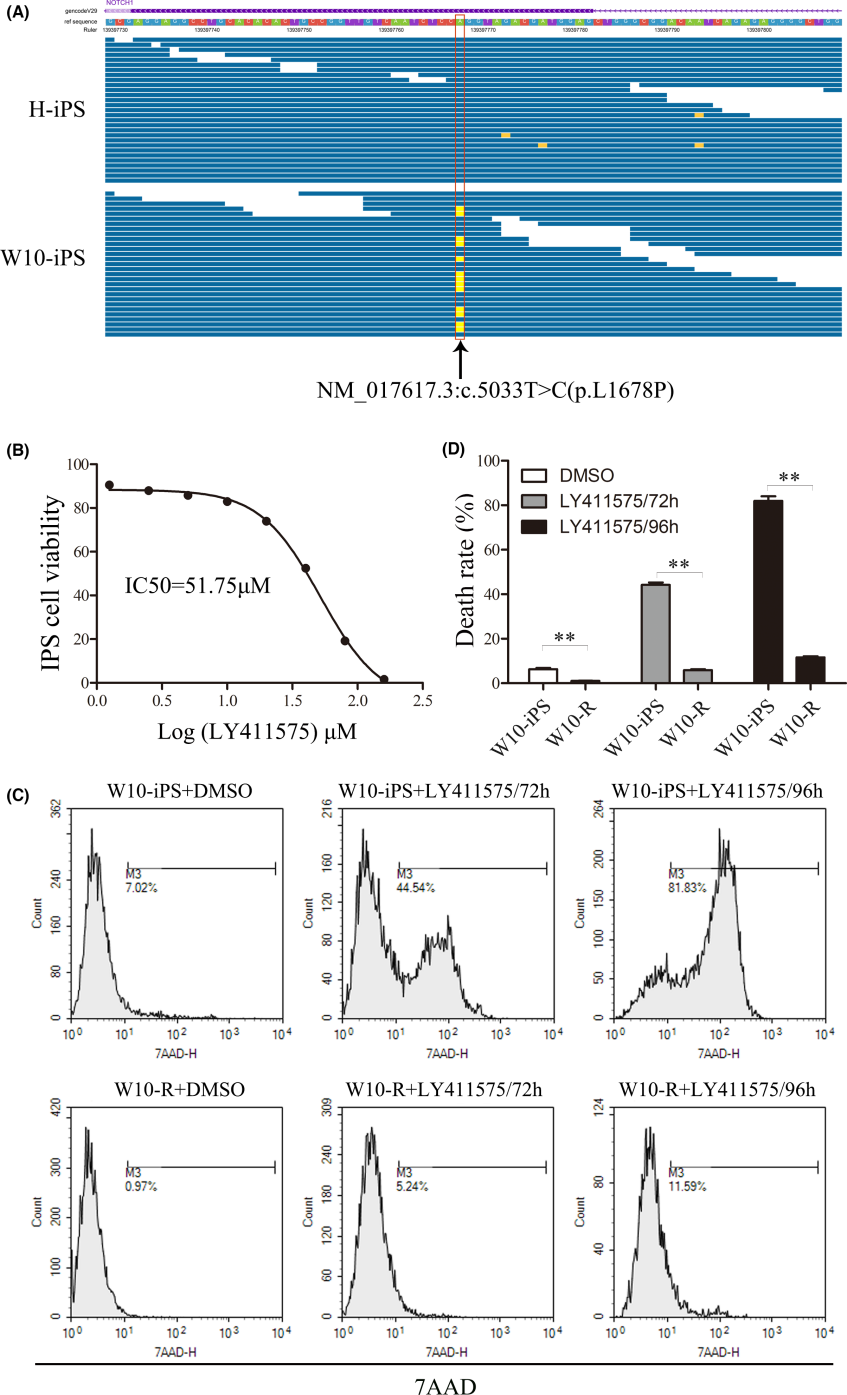
Development of LY411575‐resistant iPSCs. (A) Genomic sequence analysis showed that T‐ALL‐derived W10‐iPS contained the initial Notch1 mutation (NM_017617.3: c.5033 T > C[p.L1678P]). Each blue line represents one read. Moreover, the Notch1 mutation site is marked in yellow and pointed with the black arrow. (B) The IC50 of LY411575 on W10‐iPS. W10‐iPS were treated with varying LY411575 dosages. (C) Cell death rate of W10‐iPS (drug‐sensitive) and W10‐R (drug‐resistant) after treatment with 80 μM LY411575 for 72 and 96 h. The analysis was performed using flow cytometry after 7AAD staining. (D) Statistical graph of death rate of W10‐iPS and W10‐R cells. **p < 0.01

One case of T‐ALL harboring Notch1 mutation (NM_017617.3:c.5033 T > C [p.L1678P]) was selected to prepare T‐iPSCs. To generate iPSCs, primary T‐ALL cells were transduced with four reprogramming factors (Sox‐2, Klf4, Oct4, and Myc) using nonintegrating Sendai virus. The representative clonal growth during the reprogramming process of T‐ALL PBMC is shown in Figure 1A. The reprogrammed T‐ALL PBMCs were then assessed for pluripotency features. Successful transformation was analyzed using alkaline phosphatase staining (Figure 1B) and the expression of pluripotency markers (Figure 1C,D). Transformed T‐ALL cells were then transplanted into immunodeficient mice to form teratomas (Figure 1E). Karyotype analysis of T‐iPSCs is shown in Figure S2. T‐iPSCs (W10_iPS) containing the initial Notch1 mutation (NM_017617.3:c.5033 T > C[p.L1678P]) were identified using Genomic sequence analysis (Figure 2A). These findings demonstrated that T‐ALL cells could be transformed into iPSCs while retaining the initial genetic alteration.

### 3.2 Development of LY411575 resistant T‐iPSCs

γ‐secretase was known as an intramembrane proteolytic enzyme, which was mainly involved in the cleavage and hydrolysis of important transmembrane proteins like Notch.^31^ LY411575 is an effective γ‐secretase inhibitor that can block the Notch signaling pathway.^32^ It has the potential to become molecular‐targeted drugs in the future T‐ALL treatment. Therefore, we used LY411575 for the screening of drug‐resistant T‐iPSCs. To calculate the half maximal inhibitory concentration (IC50) of LY411575, T‐iPSCs were treated with a multiplicity increase in dosage of LY411575 ranging from 1.25 to 160 μM. Then, the rate of cell death was detected using 7AAD staining. The IC50 was found to be 51.75 μM (Figure 2B). LY411575 resistant W4‐iPS (W4‐R) and W10‐iPS (W10‐R) which could tolerate 80 μM LY411575 were established. Flow cytometry revealed that the death rates of W10‐R cells after 72 and 96 h of LY411575 treatment were (5.90 ± 0.64) % and (11.62 ± 0.89) %, respectively, lower than those of W10‐iPS cells ([44.18 ± 1.97] % and [81.97 ± 3.61] %) (Figure 2C,D).

### 3.3 *MAEL* promotes drug resistance in T‐iPSCs

**FIGURE 3:**
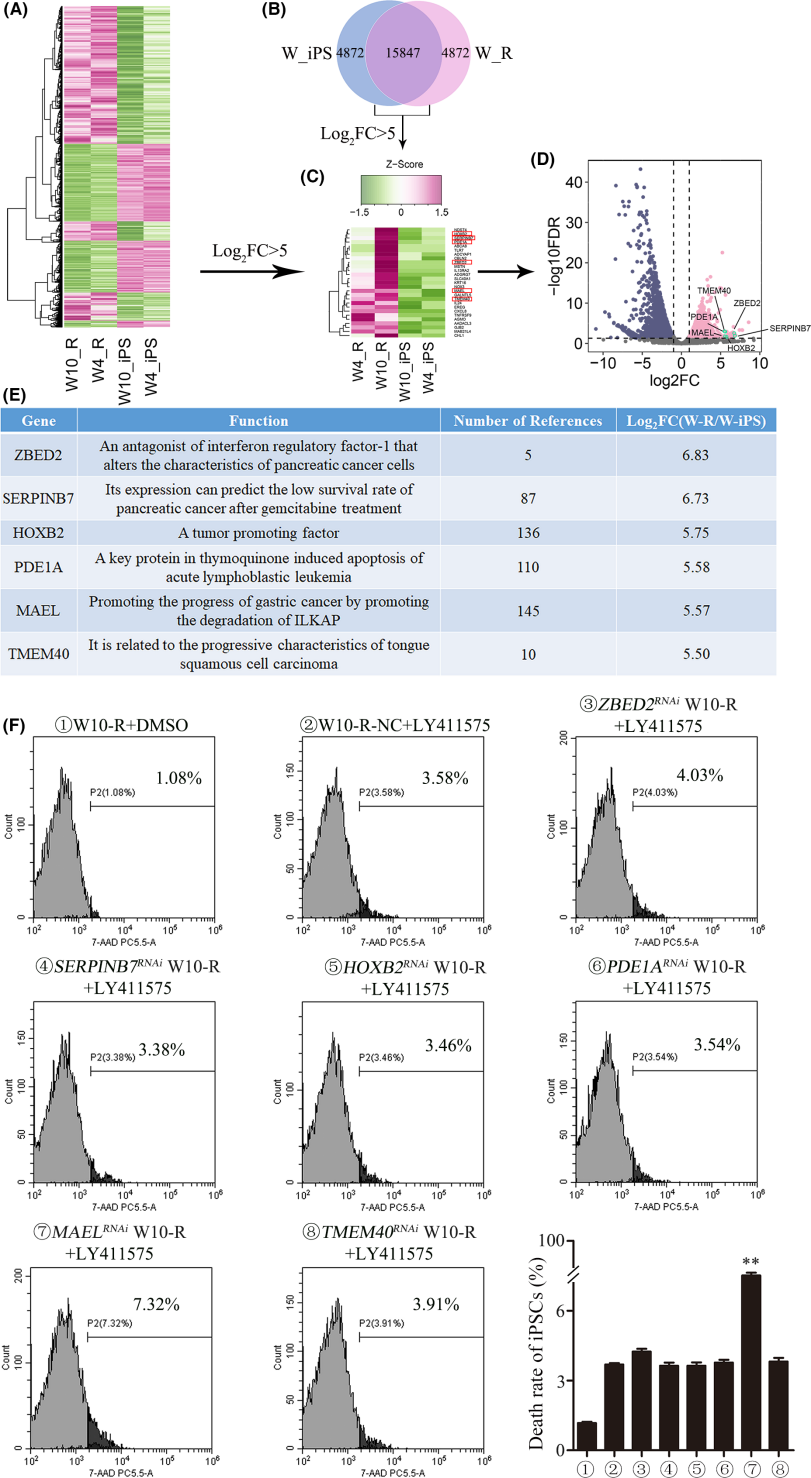
The effect of *MAEL* on drug resistance of T‐ALL‐derived iPSCs. (A) Heat map for the W_iPS (drug‐sensitive) and W_R (drug‐resistant) groups. (B) Venn diagram for the differently expressed genes between the W_iPS and W_R groups. (C) The Log_2_FC values of all differently expressed genes between W_iPS and W_R groups. The Log_2_FC value of the genes displayed in this figure was >5. (D) Volcano map of the differently expressed genes between the W_iPS and W_R groups. (E) The six most differentially expressed genes between the W_iPS and W_R groups. Gene functions and Log_2_FC values of the genes are listed. (F) W10‐R iPSCs were transfected with three pair siRNAs of six genes separately for 72 h and subsequently treated with 80 μM LY411575 for 72 h. The rate of cell death was detected using flow cytometry after 7AAD staining

Genomic analyses revealed 4872 differently expressed genes (DEGs) between the W_iPS (drug‐sensitive) and W_R (drug‐resistant) groups (Figure 3A,B). Among them, six genes including *ZBED2*, *SERPINB7*, *HOXB2*, *PDE1A*, *MAEL,* and *TMEM40* were found to be over‐expressed in the W_R group (Figure 3C,D). The specific functions and Log_2_FC values of the six genes are listed in Figure 3E. The expression levels of the six genes in W10‐iPS and W10‐R cells were shown in Figure S3. In order to identify the key regulatory genes, duplicated siRNAs against above six genes were designed to carry out loss function analysis with W10‐R iPSCs. FAM‐labeled scrambled siRNA was transfected into W10‐R iPSCs as a negative control (W10‐R‐NC) (Figure S4A). After 80 μM LY411575 treatment for 72 h, *MAEL* knockdown resulted in highest mortality (7.51 ± 0.21%) in W10‐R iPSCs (Figure 3F), implying that *MAEL* is the most important gene regulating drug resistance in T‐iPSCs.

### 3.4 *MAEL* ‐promoted drug resistance by increasing the expression of *MRP* and *LRP* genes

**FIGURE 4:**
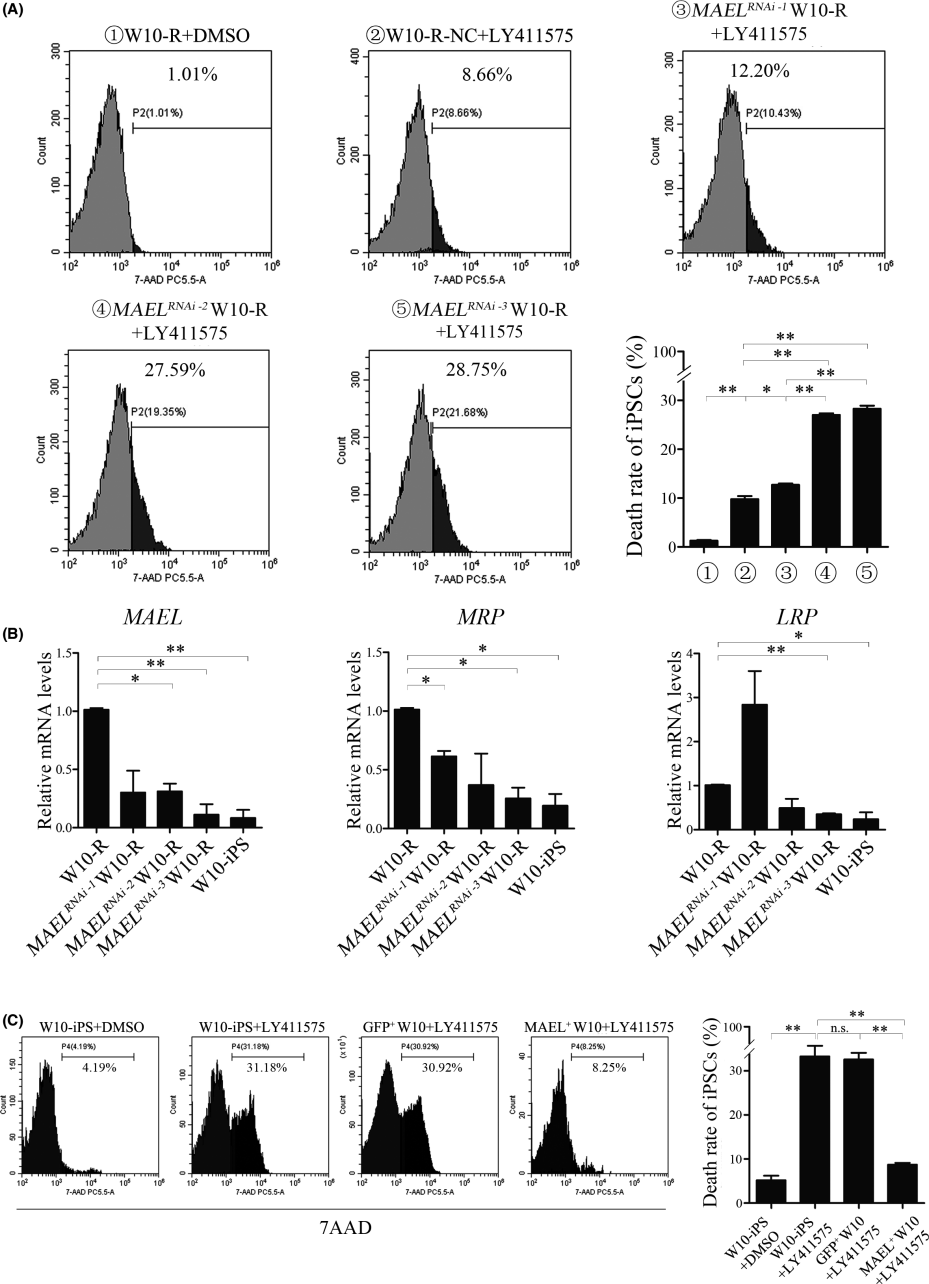
*MAEL* promoted drug resistance by increasing the expression of drug resistance genes. (A) The effect of *MAEL* knockdown on the death rate of W10‐R iPSCs under 80 μM LY411575 treatment. *MAEL* ^*RNAi‐1*^ W10‐R (③) represents MAEL‐944/1067 siRNA knockdown W10‐R cells, *MAEL* ^*RNAi‐2*^ W10‐R (④) represents MAEL‐1067/1148 siRNA knockdown W10‐R cells, and *MAEL* ^*RNAi‐2*^ W10‐R (⑤) represents MAEL‐1148/944 siRNA knockdown W10‐R cells. **p < 0.01. (B) Relative expression levels of *MAEL*, *MRP*, and *LRP* in W10‐R, *MAEL* knockdown W10‐R, and W10‐iPS cells. *p < 0.05, **p < 0.01. (C) The rate of death of W10‐iPS and MAEL‐GFP positive W10‐iPS cells. The analysis was performed using flow cytometry after 7AAD staining. **p < 0.01, ns: no significance

To further assess the relationship between the *MAEL* gene and drug resistance, we analyzed the expression of two drug resistance‐related genes (*MRP* and *LRP*) after *MAEL* knockdown. The MAEL‐944/1067 siRNA knockdown W10‐R cells were labeled as *MAEL* ^*RNAi‐1*^ W10‐R (③), MAEL‐1067/1148 siRNA knockdown W10‐R cells were labeled as *MAEL* ^*RNAi‐2*^ W10‐R (④), and MAEL‐1148/944 siRNA knockdown W10‐R cells were labeled as *MAEL* ^*RNAi‐2*^ W10‐R (⑤). Flow cytometry results showed that compared with the negative control group (②), the cell death rate was significantly high in groups ③, ④, and ⑤ and was highest in groups ④ and ⑤ (Figure 4A).

To clarify the expression of drug resistance‐related genes in W10‐R cells after the knockdown of *MAEL* gene, we assessed the relative expression of *MAEL*, *MRP*, and *LRP* genes in each group using qRT‐PCR. Compared with the control group (W10‐R), the expression of *MAEL* significantly decreased in the *MAEL*‐knockdown W10‐R cells (*MAEL* ^*RNAi‐1*^ W10‐R, *MAEL* ^*RNAi‐2*^ W10‐R, and *MAEL* ^*RNAi‐3*^ W10‐R). Meanwhile, that of *MRP* significantly decreased in the *MAEL* ^*RNAi‐1*^ W10‐R, and *MAEL* ^*RNAi‐3*^ W10‐R cells, whereas that of *LRP* significantly decreased in *MAEL* ^*RNAi‐3*^ W10‐R cells (Figure 4B, Figure S4B). These findings further demonstrated that *MAEL* knockdown downregulated the expression of *MRP* and *LRP* in W10‐R cells. Interestingly, this phenomenon partially restored the sensitivity of W10‐R cells to LY411575.

To further verify the relationship between *MAEL* gene and drug resistance, MAEL protein was over‐expressed in W10‐iPS cells. As shown in Figure 4C, the toxicity of 80 μM LY411575 on W10‐iPS overexpressing MAEL was (8.71 ± 0.41)%, significantly lower than on W10‐iPS cells (33.28 ± 2.42)% (*p* < 0.01) and GFP^+^ W10‐iPS cells (32.57 ± 1.50)% (*p* < 0.01). These results demonstrated that *MAEL* gene enhances resistance to LY411575 by upregulating the expression of drug resistance‐related genes *MRP* and *LRP*.

## 4 DISCUSSION

Chemical drugs applied in T‐ALL treatment are broad‐spectrum anticancer drugs which can suppress cell proliferation with vigorous division. These drugs have poor selectivity and significant clinical side effects. LY411575 was an effective γ‐secretase inhibitor which can block Notch signaling pathway.^32^ It has the potential to become molecular‐targeted drugs in the future T‐ALL treatment. Therefore, we used LY411575 to analyze drug resistance in T‐ALL. In this study, we successfully developed T‐ALL‐derived iPSCs (T‐iPSCs) containing the Notch 1 mutation and LY411575 resistant T‐iPSCs. Bioinformatics analyses revealed that six genes including *ZBED2*, *SERPINB7*, *HOXB2*, *PDE1A*, *MAEL,* and *TMEM40* were over‐expressed in drug‐resistant T‐iPSCs. Functional analyses implicated *MAEL* gene for drug resistance of T‐iPSCs against LY411575. On the other hand, *MAEL* knockdown downregulated the expression of *MRP* and *LRP* in drug‐resistant T‐iPSCs, which partially restored the sensitivity of the cells to LY411575.

Patient‐derived iPSCs were important study models of disease. It had several distinct advantages in pathogenesis and drug sensitivity researches.^18^, ^19^, ^20^ Our study developed a T‐ALL‐derived iPSC‐based disease model, which could be applied to pathogenesis, therapeutic drug development, drug resistance mechanism research, and guide the clinical treatment in future.

The cancer‐testis gene *MAEL* had carcinogenic effects in liver cancer, bladder cancer, colorectal cancer, gastric cancer, glioblastoma, invasive breast cancer, and lung adenocarcinoma,^33^, ^34^, ^35^, ^36^ but exerts an opposite effect on ovarian cancer.^37^ *MAEL* promotes tumor growth by inhibiting the apoptosis of cells.^38^ *MAEL* promotes tumorigenesis of gastric cancer by inducing the degradation of integrin‐linked kinase‐associated phosphatase (ILKAP).^36^ ILKAP is a serine/threonine (S/T) phosphatase and a member of the protein phosphatase 2C (PP2C) family. It played key roles in the regulation of cell survival and apoptosis.^39^, ^40^, ^41^, ^42^ In this study, we found that overexpression of *MAEL* promotes drug resistance in T‐ALL by increasing the expression of drug resistance‐related genes. Accordingly, *MAEL* is potentially a new treatment target for drug‐resistant T‐ALL.

## CONFLICT OF INTEREST

No conflict of interest to report.

## ETHICS STATEMENT

The study was approved by Ethics Committee of Shenzhen Luohu People's Hospital and conducted in accordance with applicable local regulations and the principles of the Declaration of Helsinki. Written informed consent was obtained from the participants for use of blood samples.

## Supporting information


Figure S1

Figure S2

Figure S3

Figure S4

## Data Availability

The data that support the findings of this study are available from the corresponding author upon reasonable request.
