# Supplementary figures and images for: Identification of MAEL as a promoter for the drug resistance model of iPSCs derived from T‐ALL
Source: Cancer Med. 2022 Apr 29;11(18):3479–90. doi: 10.1002/cam4.4712 (PMC9487874; doi:10.1002/cam4.4712)

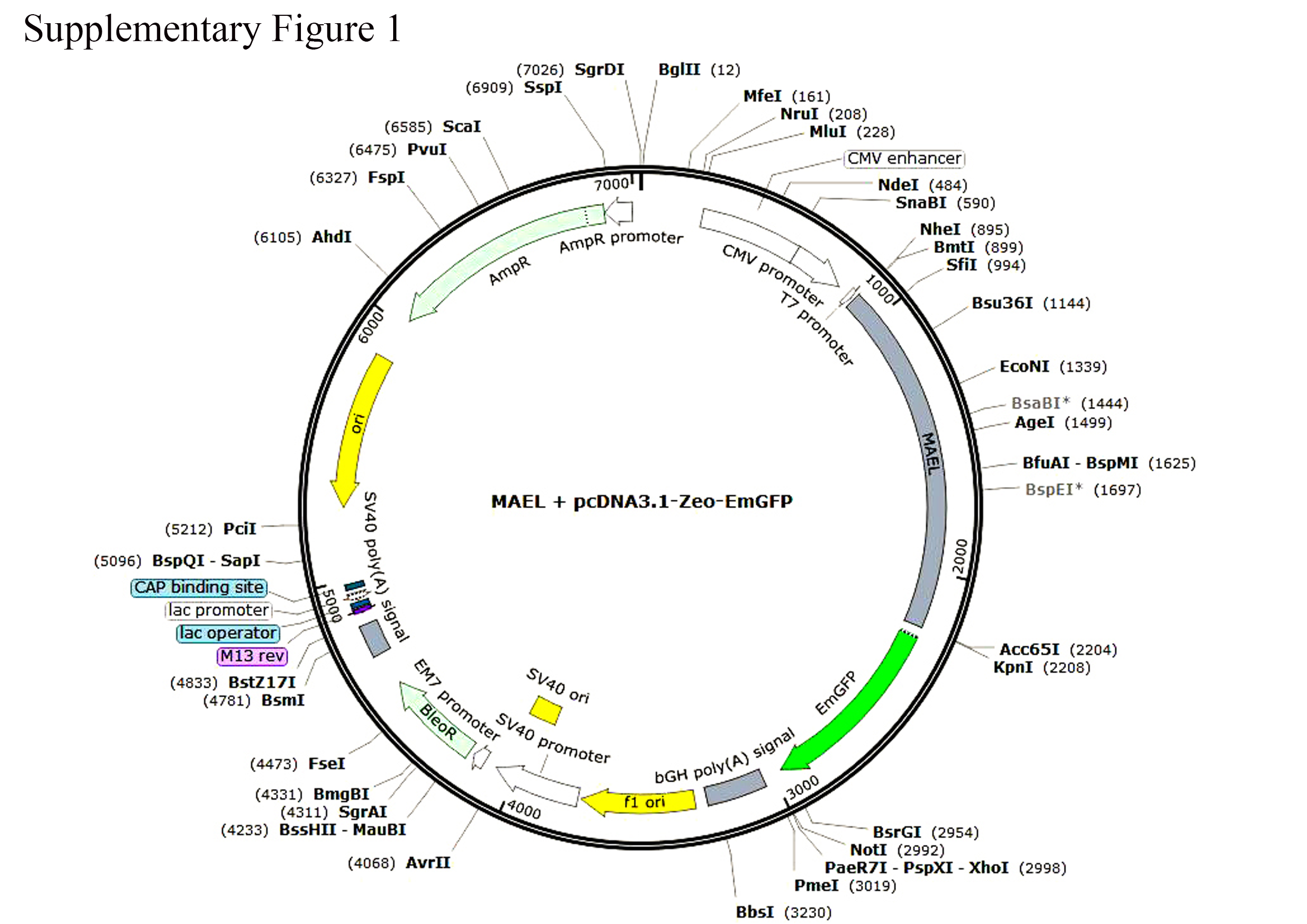

Supplement: Supplementary file 1 — Figure S1 [file CAM4-11-3479-s004.tif]

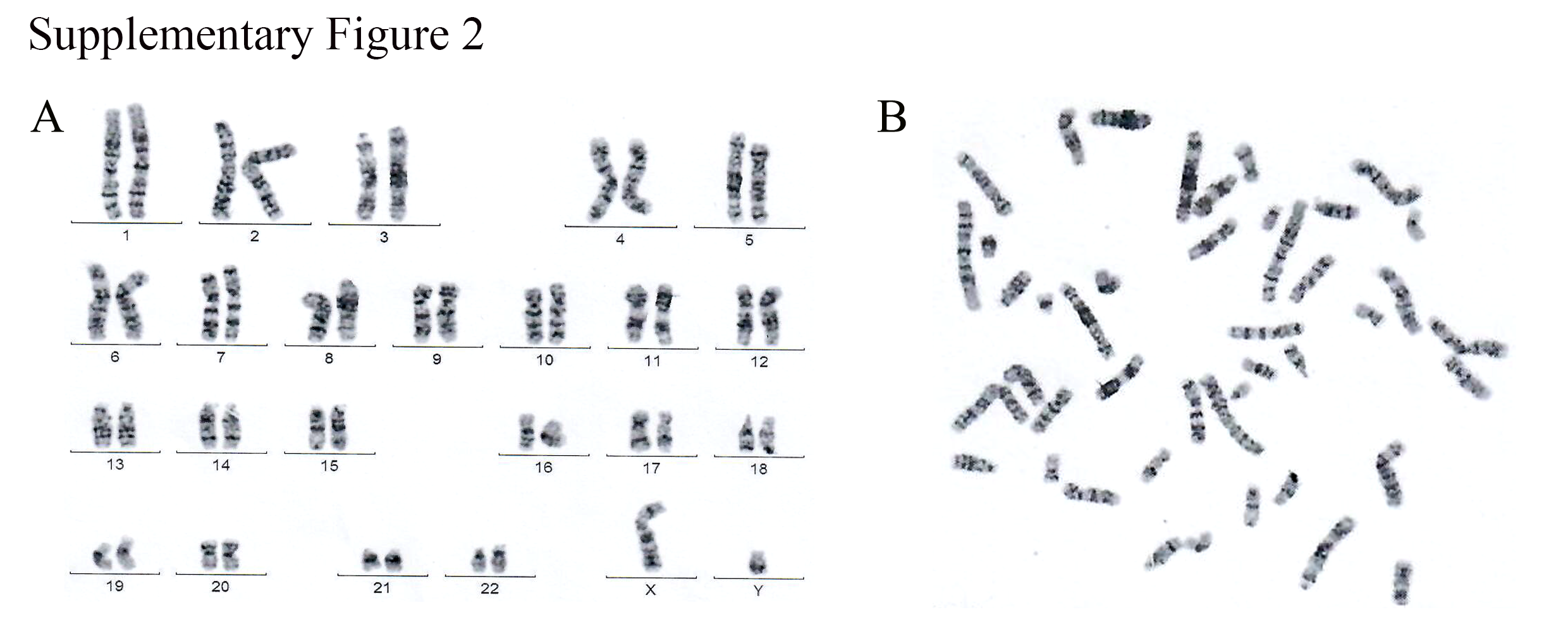

Supplement: Supplementary file 2 — Figure S2 [file CAM4-11-3479-s003.tif]

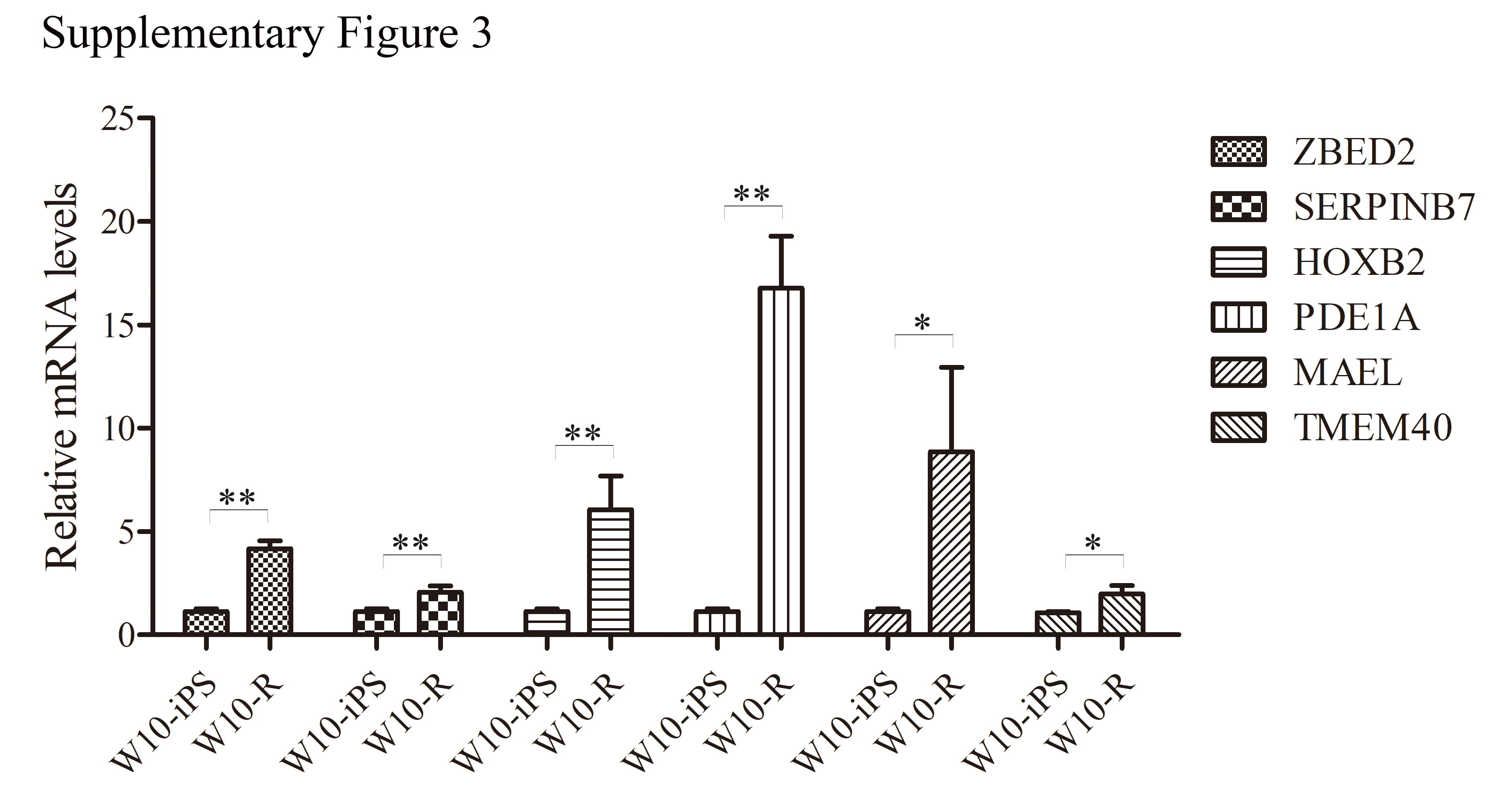

Supplement: Supplementary file 3 — Figure S3 [file CAM4-11-3479-s001.tif]

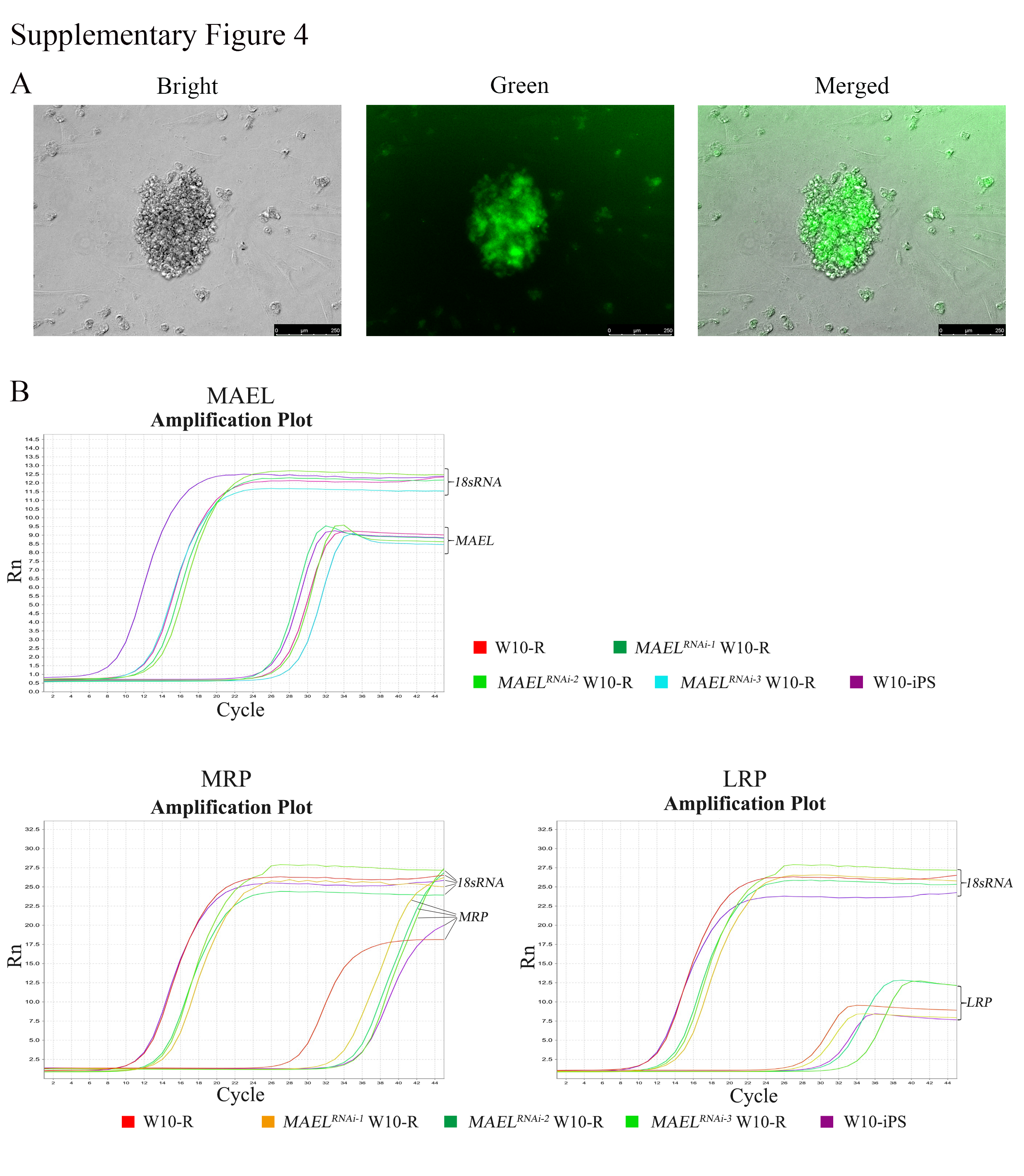

Supplement: Supplementary file 4 — Figure S4 [file CAM4-11-3479-s002.tif]
